# The Seroprevalence of *Chlamydia* Infection in Sheep in Shanxi Province, China

**DOI:** 10.3390/vetsci9120656

**Published:** 2022-11-24

**Authors:** Chen-Xu Li, Jin Gao, Sheng-Rong Shi, Wen-Wei Gao, Xing-Quan Zhu, Yu-Ping Lei, Yu Zhang, Wen-Bin Zheng

**Affiliations:** 1 College of Veterinary Medicine, Shanxi Agricultural University, Jinzhong 030801, China; 2 Key Laboratory of Veterinary Public Health of Higher Education of Yunnan Province, College of Veterinary Medicine, Yunnan Agricultural University, Kunming 650201, China; 3 Veterinary Laboratory, Shanxi Provincial Animal Disease Prevention and Control Center, Taiyuan 030008, China

**Keywords:** *Chlamydia*, *Chlamydia abortus*, seroprevalence, sheep, China

## Abstract

**Simple Summary:**

Chlamydiosis is an important zoonotic disease, which can cause significant harm and economic losses to animal husbandry. So far, there are 15 species in the genus *Chlamydia*, some of which can infect humans and many mammals including sheep, yaks, pigs, equine animals, and some wild animals. In this study, the indirect hemagglutination assay (IHA) and indirect enzyme-linked immunosorbent assay (ELISA) were used to conduct an epidemiological survey of *Chlamydia* and *Chlamydia abortus* (*C. abortus*) infection in sheep in Shanxi Province, China. The results showed that the overall seroprevalence of *Chlamydia* infection in sheep in Shanxi Province was 35.67%, and the seroprevalence was associated with the geographical location and management mode. This study provides baseline information for the prevention and control of *Chlamydia* infection in sheep in Shanxi Province, China.

**Abstract:**

*Chlamydia*, an obligate intracellular bacterium, can cause chlamydiosis in humans and animals worldwide and also leads to serious economic losses to the sheep industry. However, the information on *Chlamydia* infection in sheep was limited in Shanxi Province, northern China. In the present study, a total of 984 serum samples of sheep were collected from 11 regions in Shanxi Province, northern China in the autumn of 2020. The antibodies against *Chlamydia* and *Chlamydia abortus* were examined by the indirect hemagglutination assay (IHA) and indirect enzyme-linked immunosorbent assay (ELISA), respectively. The result showed that 351 (35.67%, 95% CI 32.68–38.66) of 984 serum samples were positive for *Chlamydia*, and the seroprevalence ranged from 6.67% to 70.79% among the different regions. In addition, antibodies to *C. abortus* infection were detected in 78 (7.93%, 95% CI 6.24–9.61) of 984 serum samples, and the seroprevalence ranged from 6.24% to 14.81% among the different regions. This is the first report on the seroprevalence of *Chlamydia* and *C. abortus* in sheep in Shanxi province, northern China. The findings provide baseline information for preventing and controlling *Chlamydia* infection in sheep in Shanxi Province, China.

## 1. Introduction

*Chlamydia* is a globally distributed obligate intracellular bacterium, which has an extensive host spectrum and can cause various clinical symptoms in humans and animals, such as endemic blindness, bacterial sexually transmitted disease, respiratory infections, and even death [1,2]. The Chlamydiaceae only has one genus, *Chlamydia*, and contains 15 species [3], and some of these pathogens, such as *C. abortus*, *C. pecorum*, *C. psittaci,* and *C. suis*, can infect sheep [4]; *C. psittaci* can cause a variety of inflammatory and respiratory symptoms, which can be life-threatening to humans and birds [5]. *C. abortus* can induce pneumonia and inflammation in sheep and is also an important cause of late-term abortion in goats and sheep, especially in the 2–3 weeks before delivery. *C. abortus* is endemic among ruminants, and sheep are the animals susceptible to *C. abortus* [6,7,8]. Although the live vaccine (*C. abortus* 1B strain) has been used for preventing the outbreaks of *C. abortus* in some regions of Europe, it still carries the risk of causing miscarriage in the sheep, and its safety remains to be clarified [9].

In pregnant women, contact with ruminants infected with *C. abortus* could cause flu-like symptoms of systemic infection, and even miscarriage [10]. Moreover, breeders and veterinarians who have more opportunities to come into contact with sheep are more likely to be infected by *C. abortus* [11]. *C. abortus* has also been widely reported in China; for example, the seroprevalence of *C. abortus* ranges from 1.76 to 15.29% in sheep in the south of China [12]. China is a large agricultural country, and animal husbandry is an important economic source for people in some regions of China. Sheep is closely related to human life, and it can provide mutton, milk, and sheepskin for humans; at the same time, sheep are also one of the most common hosts of *Chlamydia*, including *C. abortus*, so the epidemiological investigation of *Chlamydia* infection in sheep has become particularly important. However, to date, information on *Chlamydia* and *C. abortus* infection in animals has been very limited in Shanxi Province, China, seriously affecting the correct assessment of chlamydiosis in economic animals in Shanxi Province. The seroprevalence of *Chlamydia* was 13.94% (95% CI: 9.66–18.22) in alpacas in Shanxi Province [13], which was the first and the only report of *Chlamydia* infection in animals in Shanxi Province.

The objective of the present study was to investigate the seroprevalence of *Chlamydia* and *C. abortus* in sheep in Shanxi Province, and to explore the risk factors associated with *Chlamydia* and *C. abortus* infection, which could provide useful data to improve the precautionary measures for preventing *Chlamydia* and *C. abortus* infection in sheep in China.

## 2. Materials and Methods

### 2.1. Ethics Approval

The experimental procedures of the study were reviewed and approved by the Experimental Animal Ethics Committee of Shanxi Agricultural University (Approval No. 2019IACUCSXAU002A01, approved on 17 June 2019). The animals were handled in accordance with good animal practice as defined by the relevant Animal Ethics Procedures and Guidelines of the People’s Republic of China.

### 2.2. Investigation Sites

**Figure 1:**
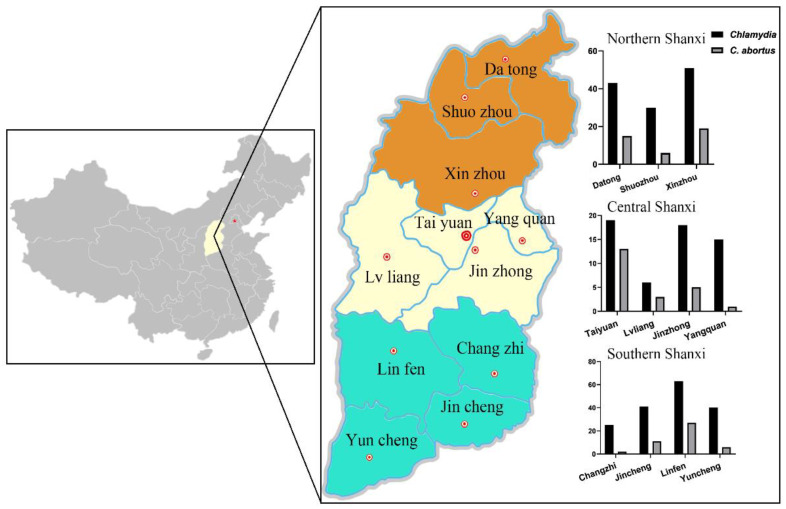
Map showing the geographical locations and seroepidemiological distribution of *Chlamydia* and *C. abortus* infection in sheep in Shanxi Province, China. Northern Shanxi: Datong City, Shuozhou City, and Xin zhou City; Central Shanxi: Taiyuan City, Yangquan City, Lvliang City, and Jinzhong City; Southern Shanxi: Linfen City, Changzhi City, Yuncheng City, and Jincheng City.

This study involved 11 regions throughout Shanxi Province, north China, including three cities located in Northern Shanxi, four cities located in Central Shanxi, and four cities located in Southern Shanxi, respectively (Figure 1). Shanxi Province (34°36′–40°44′ N, 110°15′–114°32′ E) is located in the north of China, which has a temperate continental monsoon climate with distinct differences in the four seasons; the temperature discrepancy between the north and the south is large, and the average temperature is 11 °C in autumn.

### 2.3. The Collection of Serum Samples

984 serum samples were randomly collected from sheep from 11 regions in Shanxi Province in the autumn of 2020, including Datong City (n = 90), Shuozhou City (n = 90), Xinzhou City (n = 90), Taiyuan City (n = 85), Lvliang City (n = 90), Jinzhong City (n = 90), Yangquan City (n = 90), Changzhi City (n = 90), Jincheng City (n = 90), Linfen City (n = 89), and Yuncheng City (n = 90); with 270, 355, and 359 serum samples being collected from Northern Shanxi, Central Shanxi, and Southern Shanxi, respectively (Table 1). At least three farms were sampled in each city and a total of 43 farms were sampled in this study. These 43 farms include 32 household animal farms (n = 682) (in each of these farms, the annual stock is no more than 300 sheep), six animal farming cooperatives (n = 120) (each farming cooperative is composed of multiple household animal farms), and five large-scale animal farming companies (n = 180) (in each of these companies, the annual stock is more than 1000 sheep) (Table 2). The serum samples were transported to the laboratory and stored at −40 °C.

### 2.4. Serological Tests

The specific antibodies against *Chlamydia* at genus level from sheep were detected by indirect hemagglutination assay (IHA) using a commercially available kit (Lanzhou Veterinary Research Institute, Chinese Academy of Agricultural Sciences, Lanzhou, China) according to the manufacturer’s instructions, and this kit can be used to detect antibodies against *Chlamydia* at genus level in all mammals [14,15,16]. Briefly, the serum was diluted by serial fourfold from 1:4 to 1:64, and the positive and negative serum were separately added to each plate. The serum samples were considered positive if agglutinated layers of erythrocytes formed in a dilution of 1:16 or higher. The results between 1:4 and 1:16 were considered as “doubtful” and were retested. The antibodies against *C. abortus* were determined by using a commercially available ELISA kit (Innovative Diagnostics, Grabels, France) in accordance with the manufacturer’s recommendations [17,18,19,20]. Before the antibody detection, the kits and serum samples were placed at room temperature (20 °C) for one hour. Samples with OD_sample_/OD_positive_ × 100% (S/P%) ≥ 60% or ≤50% were positive or negative, respectively, and 50 < S/P% < 60% were considered as “doubtful” and were retested.

### 2.5. Statistical Analyses

A chi-square test was performed for seroprevalence and potential risk factors (geographic location and management mode) of *Chlamydia* and *C. abortus* infection in sheep in Shanxi Province, China using SPSS 26.0 software (Chicago, IL, USA). The odds ratios (ORs) and the 95% confidence interval (95% CI) of each factor were analyzed in this study; *p* value < 0.05 was considered statistically significant.

## 3. Results

In this study, antibodies to *Chlamydia* infection were detected in 351 (35.67%, 95% CI 32.68–38.66) of the 984 sheep serum samples by IHA, with the seroprevalence ranging from 6.67% to 70.79% among the different regions (Table 1). Furthermore, the geographical location (*p* < 0.01) and management mode (*p* < 0.05) were revealed as risk factors for *Chlamydia* infection in sheep in Shanxi Province (Table 2).

In this study, antibodies to *C. abortus* infection were detected in 78 (7.93% 95% CI 6.24–9.61) of the 984 sheep serum samples by ELISA with the seroprevalence ranging from 1.11% to 21.11% among the different regions (Table 3). Moreover, geographical location (*p* < 0.01) was revealed as a risk factor for *C. abortus* infection in sheep in Shanxi Province (Table 4).

## 4. Discussion

In the present study, the seroprevalence of *Chlamydia* infection in sheep in Shanxi Province was 35.67% based on IHA (Table 1), which was higher than that in black-boned sheep in Yunnan Province, China (22.76%) [16], but lower than that in sheep in Shandong Province, China (52.78%) [10]. The seroprevalence of *Chlamydia* infection in sheep has been widely reported around the world, such as 15.23% in sheep in Germany [21], and 8.9% in sheep in India [22]. Furthermore, the seroprevalence of *C. abortus* in sheep in Shanxi Province was 7.93% based on ELISA (Table 3), which was lower than that in most provinces in China; for example, it is slightly lower than that in goats in Hunan Province (8.45%) [23], and also lower than that in sheep in Gansu Province (18.65%) [8]. The seroprevalence of *C. abortus* infection also has been reported in sheep worldwide, such as 7.98% in sheep in southern Ethiopia [24], and 19.2% in sheep in Australia [25]. The different seroprevalences may be due to the living environment, geographic location, and climatic conditions.

The distance between the northern Shanxi Province and the southern Shanxi Province is more than 680 km, and the large temperature differences and different local conditions and customs may affect the infection of animals with different pathogens. In the present study, the seroprevalence of *Chlamydia* in sheep was different in different regions, ranging from 6.76% in Lvliang City located in Central Shanxi, to 56.67% in Xinzhou City located in Northern Shanxi, and 70.79% in Linfen City located in Southern Shanxi (Table 1). The geographical location was revealed as a risk factor for *Chlamydia* infection in sheep in Shanxi Province (*p* < 0.01) in that higher seroprevalence of *Chlamydia* infection in sheep was detected in Northern and Southern Shanxi, and lower seroprevalence was detected in Central Shanxi (Table 2). Compared with sheep in Central Shanxi (16.34%), sheep in Northern Shanxi (45.93%) and Southern Shanxi (47.08%) had more than four times (OR = 4.55, 95% CI = 3.21–6.46) and nearly four times (OR = 3.86, 95% CI = 2.66–5.59) higher risk of acquiring *Chlamydia* infection, respectively. These results indicated that sheep had the lowest risk of acquiring *Chlamydia* infection in Central Shanxi. Compared with Southern and Northern Shanxi, the temperature and humidity in Central Shanxi are at the middle level, but the seroprevalence of *Chlamydia* was lower than that in Southern and Northern Shanxi, so the results indicated that climatic conditions do not seem to be the main factor affecting *Chlamydia* infection in sheep in Shanxi Province.

In order to explore the possible causes of this phenomenon, the seroprevalence of *Chlamydia* infection in sheep under different management modes was analyzed. The results showed that compared with sheep in animal farming cooperatives (24.17%), sheep in household animal farms (37.72%) and large-scale animal farming companies (35.56%) had more than two times (OR = 2.48, 95% CI = 1.59–3.89) and nearly two times (OR = 1.73, 95% CI = 1.32–2.91) higher risk of acquiring *Chlamydia* infection, respectively. These results indicated that management mode was a risk factor for *Chlamydia* infection in sheep in Shanxi Province (*p* < 0.05). The capital and the core area of economic development of Shanxi Province are located in Central Shanxi which has led the economic development of Shanxi Province. In animal husbandry, the management mode of animal farming cooperatives with standardized management has been established in Central Shanxi, which may be the main reason for the lower seroprevalence of *Chlamydia* infection in sheep in Central Shanxi.

The Chlamydiaceae family only has one genus but contains 15 species [3], and different species of *Chlamydia* may have different transmission characteristics. To explore the seroprevalence of *C. abortus* in sheep in Shanxi province, the antibodies against *C. abortus* were tested by ELISA. A total of 78 of 984 (7.93%, 95% CI 6.24–9.61) examined sheep serum samples were positive for *C. abortus* infection, accounting for 22.22% (78/351) of all *Chlamydia* infections in this study. *C. abortus* infection in sheep was distributed in all examined cities in Shanxi Province with different seroprevalences, ranging from 1.11% to 21.11% (Table 3). Xinzhou City (21.11%) and Datong City (16.67%) located in Northern Shanxi had higher *C. abortus* seroprevalence in sheep, which was consistent with the infection trend of *Chlamydia* in Shanxi Province. However, in some regions, very low levels of *C. abortus* infection in sheep was detected, such as in Changzhi City (1.11%) and Yuncheng City (1.11%) located in Southern Shanxi, and Yangquan (1.11%) located in Central Shanxi, which was quite different from the overall *Chlamydia* prevalence in these regions, suggesting that *C. abortus* may not be the dominant species of *Chlamydia* infection in sheep in these regions, which warrants further investigation. By analyzing the potential risk factors (geographical location and management mode) of *C. abortus* infection, only the geographical location was revealed as a risk factor for *C. abortus* infection in sheep in Shanxi Province (*p* < 0.01) (Table 4). Among these three geographical locations, sheep in Southern Shanxi (5.93%, 95% CI 3.11–8.74) had the lowest seroprevalence of *C. abortus*, which was similar to sheep in Central Shanxi (6.20%, 95% CI 3.69–8.71), but far lower than that in Northern Shanxi (14.80%, 95% CI 10.58–19.52). Sheep in Northern Shanxi had approximately four times (OR = 3.72, 95% CI = 2.04–6.82) higher risk of acquiring *C. abortus* infection than that in Southern Shanxi.

The overall seroprevalence of *Chlamydia* infection in sheep in Shanxi Province was 35.67%, while the seroprevalence of *C. abortus* infection was only 7.93%. The difference was more obvious in Southern Shanxi (47.08% versus 5.93%). These results suggested that *C. abortus* is not the dominant species of *Chlamydia* infection in sheep in Shanxi Province. According to the result of another study, the main *Chlamydia* species infecting sheep are *C. abortus* and *C. pecorum* [26]; therefore, it is likely *C. pecorum* is the dominant species of *Chlamydia* in Shanxi Province. However, more studies are needed to detect the main species of *Chlamydia* in sheep in Shanxi Province.

Overall, both *Chlamydia* and *C. abortus* infections appear to be severe in sheep in Northern Shanxi, and low temperatures in Northern Shanxi may facilitate the long-term survival of *Chlamydia* in the environment, including *C. abortus* [27]. In addition, most areas of Shanxi Province lie between the Lvliang Mountains and Taihang Mountains which isolate Shanxi Province from Shaanxi, Hebei, and Henan Provinces; only the northern part of Shanxi Province directly borders the Inner Mongolia grasslands, and the living habits are closer to the people in Inner Mongolia Autonomous Region (IMAR). The seroprevalence of *Chlamydia* in pigeons in Tongliao City of IMAR was 35.14%, which could facilitate the spread of *Chlamydia* to susceptible animals through direct contact or fecal contamination [28], and the seroprevalence of *C. abortus* in commercial dairy and beef cattle in IMAR was 12.96% [29]. According to the above results and the actual situation in Shanxi Province (the production type is mainly mutton), in addition to improving the management mode in the sheep industry, it is also imperative to strengthen the prevention and control of animal diseases between the boundaries to avoid cross-infection. In addition, a limitation of the present study is that more potential risk factors, such as gender, age, other co-infections, or vaccination programs, were not included in the analysis, because such information was incomplete and could not be unified among the 43 sampling sites. Further analysis of other potential risk factors and the continuous detection of *Chlamydia* infection in sheep herds are the directions of our future efforts.

## 5. Conclusions

The present investigation revealed that the overall seroprevalence of *Chlamydia* infection in sheep in Shanxi Province is high (35.67%, 351/984), and the seroprevalence of *C. abortus* infection in sheep in this province is 7.93%. Geographical location and management mode were the risk factors associated with *Chlamydia* infection in sheep in Shanxi Province, China. This is the first report of the seroprevalence of *Chlamydia* and *C. abortus* in sheep in Shanxi Province, which provides baseline data for the prevention and control of *Chlamydia* and *C. abortus* infection in sheep in Shanxi Province.

## Figures and Tables

**Table 1:** The seroprevalence of *Chlamydia* infection in sheep in different cities of Shanxi Province, China.

Geographical Location	City	No. Examined	No. Positive	Prevalence (%)
Northern Shanxi	Datong Shuozhou Xinzhou	90 90 90	43 30 51	47.78 33.33 56.67
Central Shanxi	Taiyuan Lvliang Jinzhong Yangquan	85 90 90 90	19 6 18 15	22.35 6.67 20.00 16.67
Southern Shanxi	Changzhi Jincheng Linfen Yuncheng	90 90 89 90	25 41 63 40	27.78 45.56 70.79 44.44
Total		984	351	35.67

**Table 2:** The seroprevalence of *Chlamydia* infection in sheep in three geographical locations and management modes in Shanxi Province, China.

Variable	Category	No. Examined	No. Positive	Prevalence (%) (95% CI)	*p*-Value	OR (95% CI)
Geographical location	Northern Shanxi Central Shanxi Southern Shanxi	270 355 359	124 58 169	45.93 (39.98–51.87) 16.34 (12.49–20.18) 47.08 (41.91–52.24)	<0.01	4.55 (3.21–6.46) Reference 3.86 (2.66–5.59)
Management mode	Household animal farms Animal farming cooperatives Large-scale animal farming companies	684 120 180	258 29 64	37.72 (34.09–41.35) 24.17 (16.51–31.83) 35.56 (28.56–42.55)	<0.05	2.48 (1.59–3.89) Reference 1.73 (1.03–2.91)
Total		984	351	35.67 (32.68–38.66)		

**Table 3:** The seroprevalence of *C. abortus* infection in sheep in different cities of Shanxi Province, China.

Geographical Location	City	No. Examined	No. Positive	Prevalence (%)
Northern Shanxi	Datong Shuozhou Xinzhou	90 90 90	15 6 19	16.67 6.67 21.11
Central Shanxi	Taiyuan Lvliang Jinzhong Yangquan	85 90 90 90	13 3 5 1	15.29 3.33 5.56 1.11
Southern Shanxi	Changzhi Jincheng Linfen Yuncheng	90 90 89 90	1 10 4 1	1.11 11.11 4.49 1.11
Total		984	78	7.93

**Table 4:** The seroprevalence of *C. abortus* infection in sheep in three geographical locations and management modes in Shanxi Province, China.

Variable	Category	No. Examined	No. Positive	Prevalence (%) (95% CI)	*p*-Value	OR (95% CI)
Geographical location	Northern Shanxi Central Shanxi Southern Shanxi	270 355 359	40 22 16	14.80 (10.58–19.52) 6.20 (3.69–8.71) 5.93 (3.11–8.74)	<0.01	3.72 (2.04–6.82) 1.42 (0.73–2.74) Reference
Management mode	Household animal farms Animal farming cooperatives Large-scale animal farming companies	684 120 180	48 10 20	7.02 (5.10–8.93) 8.33 (3.39–13.28) 11.11 (6.52–15.70)	>0.05	Reference 1.21 (0.59–2.45) 1.66 (0.96–2.87)
Total		984	78	7.93 (6.24–9.61)		

## Data Availability

All datasets generated for this study are included in the article.
